# Transcriptomics and network analysis highlight potential pathways in the pathogenesis of pterygium

**DOI:** 10.1038/s41598-021-04248-x

**Published:** 2022-01-07

**Authors:** Juliana Albano de Guimarães, Bidossessi Wilfried Hounpke, Bruna Duarte, Ana Luiza Mylla Boso, Marina Gonçalves Monteiro Viturino, Letícia de Carvalho Baptista, Mônica Barbosa de Melo, Monica Alves

**Affiliations:** 1 Department of Ophthalmology and Otorhinolaryngology, School of Medical Sciences, University of Campinas (UNICAMP), Rua Tessália Vieira de Camargo. Cidade Universitária, Campinas, São Paulo 13083887 Brazil; 2 Bone Metabolism Laboratory, FMUSP, University of Sao Paulo, São Paulo, SP Brazil; 3 Center for Molecular Biology and Genetic Engineering, University of Campinas (UNICAMP), Campinas, SP Brazil

**Keywords:** Computational biology and bioinformatics, Genetics, Medical research, Pathogenesis

## Abstract

Pterygium is a common ocular surface condition frequently associated with irritative symptoms. The precise identity of its critical triggers as well as the hierarchical relationship between all the elements involved in the pathogenesis of this disease are not yet elucidated. Meta-analysis of gene expression studies represents a novel strategy capable of identifying key pathogenic mediators and therapeutic targets in complex diseases. Samples from nine patients were collected during surgery after photo documentation and clinical characterization of pterygia. Gene expression experiments were performed using Human Clariom D Assay gene chip. Differential gene expression analysis between active and atrophic pterygia was performed using limma package after adjusting variables by age. In addition, a meta-analysis was performed including recent gene expression studies available at the Gene Expression Omnibus public repository. Two databases including samples from adults with pterygium and controls fulfilled our inclusion criteria. Meta-analysis was performed using the Rank Production algorithm of the RankProd package. Gene set analysis was performed using ClueGO and the transcription factor regulatory network prediction was performed using appropriate bioinformatics tools. Finally, miRNA-mRNA regulatory network was reconstructed using up-regulated genes identified in the gene set analysis from the meta-analysis and their interacting miRNAs from the Brazilian cohort expression data. The meta-analysis identified 154 up-regulated and 58 down-regulated genes. A gene set analysis with the top up-regulated genes evidenced an overrepresentation of pathways associated with remodeling of extracellular matrix. Other pathways represented in the network included formation of cornified envelopes and unsaturated fatty acid metabolic processes. The miRNA-mRNA target prediction network, also reconstructed based on the set of up-regulated genes presented in the gene ontology and biological pathways network, showed that 17 target genes were negatively correlated with their interacting miRNAs from the Brazilian cohort expression data. Once again, the main identified cluster involved extracellular matrix remodeling mechanisms, while the second cluster involved formation of cornified envelope, establishment of skin barrier and unsaturated fatty acid metabolic process. Differential expression comparing active pterygium with atrophic pterygium using data generated from the Brazilian cohort identified differentially expressed genes between the two forms of presentation of this condition. Our results reveal differentially expressed genes not only in pterygium, but also in active pterygium when compared to the atrophic ones. New insights in relation to pterygium’s pathophysiology are suggested.

## Introduction

Pterygium is a common disease of the ocular surface, consisting of a fibrovascular tissue that arises from the conjunctiva and extends to the cornea. It is related to irritative symptoms, poor aesthetics, astigmatism and, sometimes, worsening visual acuity, being a condition frequently associated with decreased quality of life. The prevalence of pterygium varies according to the region studied, being higher in tropical regions, where the prevalence is approximately 22%, and lower in countries outside these areas, where it is close to 2%^1,2^. In Brazil, the reported rate of pterygium in a city of the Southwest region was 8.12%^3^, while in indigenous populations of the Brazilian Amazon rainforest, the prevalence varied from 18.4 to 36.6%^4^. Several risk factors for pterygium have been listed, although the most frequent ones are exposure to ultraviolet radiation, chronic irritation and inflammation^5–7^. Surgery is considered for symptomatic and cosmetic improvement, but recurrence can happen in 0 to 88% of the cases, depending on the surgical technique employed^8^. The development of the pterygium has some similarities with tumor growth, such as fibrovascular proliferation, corneal invasion and high recurrence rate after surgical excision^9^. Among the mechanisms and structures involved, it is known that the proliferation and degeneration of collagen fibers, especially type I collagen, plays an important role in the development of the pterygium^10^. Several studies also demonstrate the role of anti-apoptotic mechanisms, modulation of extracellular matrix under ultraviolet radiation exposure, angiogenesis, inflammation and hereditary factors in the pathogenesis of pterygium^11–13^. However, the exact pathogenesis is not yet fully understood and the precise identity of the critical triggers as well as the hierarchical relationship between all the elements involved in the pathogenesis of this disease are yet to be described.

In view of the controversies on the multiple mechanisms of pterygium formation, studies were carried out using high-throughput genomic technologies, such as microarrays, to attempt to elucidate mRNA expression patterns that could underlie specific molecular events of interest in its pathogenesis^14,15^. Also, several other studies analyzed non-coding RNA expression, evidencing upregulated and downregulated miRNAs and lncRNAs that could be involved in pterygium pathogenesis^12,16–25^. Microarray-based studies generate large databases of raw gene expression that are deposited in data repositories for public reuse. In turn, meta-analysis of these data may enhance results and generate new biological insights regarding some conditions in comparison with individual studies, once it reduces study biases, increases statistical power and obtains a more accurate evaluation of differentially expressed genes (DEGs).

Over the past decade, the availability of large public genomic datasets and robust bioinformatics tools enabled the development of new data mining approaches in biomedical research^26^. Consequently, the field of integrative analysis of high throughput gene expression datasets has grown exponentially, leading to the development of tools and guidance for conducting a meta-analysis of gene expression^14,27,28^. Meta-analysis of transcriptomic datasets deposited in public repositories represents an attractive strategy capable of identifying key pathogenic mediators and therapeutic targets in complex diseases^14,29,30^. By combining multiple study datasets, this strategy has the potential to increase the statistical power and to generate new biological insights that could not be accessed from original studies^14^. Moreover, several bioinformatics tools of curated biological databases^31–33^ have been recently developed, making even easier the interpretation of relevant differentially expressed (DE) genes sets identified from meta-analysis and the prediction of associated pathways and regulatory networks^29,31,32,34^. In this context, a well conducted meta-analysis has the potential to contribute to the generation of new hypothesis about the pathogenic mechanisms of complex diseases.

The aims of this study were to evaluate gene expression in pterygium samples, perform a meta-analysis with two databases available at the Gene Expression Omnibus public repository and then, correlate the findings through a miRNA-mRNA regulatory network.

## Methods

### Microarray data analysis of the Brazilian cohort

#### Sample collection

The present study was carried out with the approval of the Research Ethics Committee of the University of Campinas (UNICAMP) and was conducted in accordance with the tenets of the Declaration of Helsinki and current legislation on clinical research. Written informed consent was obtained from all subjects after explanation of the procedures and study requirements.

Participants were selected by convenience, at the Ocular Surface outpatient clinic of the Hospital of University of Campinas. Participants that presented nasal pterygia, not previously operated, and wanted surgery were included. Individuals with conjunctival surface diseases such as previous chemical burns, pemphigoid and cicatricial diseases, or suspected ocular surface neoplasia were excluded.

The pterygium was classified according to the fibrovascular tissue extension towards the cornea (grade 1 when the lesion reaches the limbus, grade 2 when it covers the cornea at about 2 mm, grade 3 when it reaches the pupil margin and grade 4 when it exceeds the pupil) and according to its biomicroscopic aspect (atrophic when the visualization of structures immediately below is possible, or active when fibrovascular tissue prevents proper visualization of underneath structures). The latter classification is based on the classification proposed by Tan et al., in which the pterygia are divided according to tissue translucency. Grade T1 (atrophic) denotes a pterygium in which episcleral vessels underlying the body of the pterygium are unobscured and clearly distinguished. Grade T3 (active or fleshy) denotes a thick pterygium in which episcleral vessels underlying the body of the pterygium are totally obscured. All other pterygia that do not fall into these 2 categories are classified as Grade 2 (intermediate). An increased fleshiness or thickness of the fibrovascular component of the pterygium is believed to be associated with recurrence after excision^35^.

Participants underwent pterygium excision followed by an upper bulbar conjunctival free autograft placed over the site of the original lesion. All pterygia specimens were nasally located. The entire pterygium tissue was collected, and the samples were immediately stored in RNAlater stabilization solution (Invitrogen) until RNA extraction.

#### Microarray assay

Tissue samples were transferred to liquid nitrogen and ground into powder. Total RNA extraction was performed using TRIzol Reagent (Invitrogen) and purified with RNeasy RNA extraction kit (Qiagen) according to the manufacturer's protocol. RNA concentration and purity were assessed through NanoDrop 2000 spectrophotometer (Thermo Scientific) whereas RNA integrity was measured in the Bioanalyzer 2100 System (Agilent Technologies). Samples with an RNA integrity number ≥ 7 were subjected to microarray analysis.

Fifty nanograms of total RNA were used to generate ss-cDNA, which was hybridized on Human Clariom D Assay gene chip (Affymetrix). This array interrogates more than 540,000 human transcripts. Sample labeling, hybridization, washing and scanning were performed according to manufacturer’s protocols at Molecular Core Facility (São Paulo, SP, Brazil).

#### Microarray data normalization and probe sets annotation

The normalization of the transcriptomic data of 9 samples from our cohort was performed by SST-RMA summarization to generate gene level expression signals using Transcriptome Analysis Console (TAC 4.0, Applied Biosystems) software after the evaluation of quality control metrics. Affymetrix probes were annotated to human genome hg38 version using TAC software and the Clariom D annotation file Clariom_D_Human.r1.na36.hg38.a1.transcript.csv. Probes mapped to more than one gene were removed and logarithm transformed expression data was exported for further analysis^33,36^.

This normalized expression data of the Brazilian cohort was later used for extracting the expression intensity of miRNAs and their respective targets to reconstruct the miRNA-mRNA regulatory network, that was based in data from the meta-analysis (described later).

#### Evaluation of DE genes comparing active vs atrophic pterygium

To identify the divergent gene expression pattern between active and atrophic pterygium, a differential gene expression analysis was performed using a linear model fitted in limma package^37^ after adjusting variables by age. We also applied the Empirical Bayes framework to estimate the more precise expression of each gene to discriminate DE genes. DE genes were identified based on the adjusted *p* valor < 0.05 and filtered with the fold change values. To evaluate the discriminatory capacity of each DE gene, we computed the area under (AUC) the ROC curve using pROC package^38^.

### Meta-analysis of gene expression studies

#### Pre-processing

Microarray raw data were pre-processed using the Robust Multichip Average (RMA) method^39^ implemented in the oligo package^40^ as previously described^29^. Briefly, RMA algorithm performs background subtraction which intends to minimize the effects of the noises inherent to microarray technology. Quantile normalization and median-polish steps are then applied to mitigate the effects of technical variables through the estimation of a common intensity distribution across samples. Probes are summarized into a single probe set corresponding to a single gene and expression is then transformed in log-scale. Probe sets were annotated using biomaRt packages^41^.

#### Identification of eligible datasets

Gene expression datasets from microarray studies including human patients that underwent pterygium excision were searched in the Gene Expression Omnibus (GEO) public repository, by December 2020. Search was conducted using the term “pterygium”. Datasets were only included if the studies analyze both affected patients and healthy tissues. All included datasets were from studies published in peer-reviewed journals. In addition, studies were excluded if samples were submitted to cell culture.

#### Meta-analysis of gene expression

The meta-analysis was performed with RankProd package^42^ using Rank product algorithm. A non-parametric method is applied to detect genes consistently ranked as DE by comparing patients to healthy controls in each dataset. Thus, this method overcomes the heterogeneity among multiple datasets by transforming the actual expression values into ranks^42^ and computes the *p* value and the false discovery rate (FDR) of each DE gene. The gene list was further filtered to include only genes that were up- or down-regulated in the same direction in both studies based on a false discovery rate (FDR) < 0.05 as previously described^29^. Heatmap visualization of the subset of 30 top DE genes was performed using the heatmap Bioconductor package^43^ after expression recalibration using a list of human housekeeping genes derived from the HRT Atlas database^44^.

#### Functional gene set analysis

To facilitate the interpretation of the biological significance of the up-regulated genes identified by the meta-analysis, a functional gene set analysis (GSA) was performed using ClueGO^45^. ClueGO is a plug-in of Cytoscape^36^, which predicts the functional gene ontology terms or biological pathways and organizes them in functionally grouped networks, highlighting the biological relationship between them. ClueGO’s fusion criterion was applied to reduce the redundancy of the terms that have similar associated gene sets. In order to filter the most important terms and pathways, we used two-sided hyper-geometric distribution tests and terms at a significant level Bonferroni adjusted p-value of ≤ 0.05.

#### Functional analysis of down-regulated genes

To gain more insights into the biological processes associated with the down-regulated signature, these genes were used for an additional functional analysis based on the Functional Analysis of Individual Microarray/RNAseq Expression (FAIME) algorithm implemented in seq2pathway package^34^. Briefly, FAIME determines at a single sample level the cumulative quantitative effects of genes inside the differentiated Gene Ontology terms and biological processes. A FAIME score is then computed based on the gene expression pattern of each sample for the predicted terms or biological processes. Receiver operating characteristic (ROC) curve was computed to evaluate the discriminatory capacity of each of these enriched terms for a binary classification. In this analysis, the binary classification was pterygium vs control, across FAIME scores computed for each pathway. We computed the area under (AUC) the ROC curve using pROC R package^38^. Finally, we selected the enriched terms with AUC equal or more than 0.7 and network visualization was performed using cytoscape software^36^.

#### Prediction of miRNA-mRNA regulatory network

The list of targeted genes included in this analysis was derived from the functional gene set analysis performed with the up-regulated genes identified by the meta-analysis. Briefly, we extracted the list of genes associated with the functional gene ontology terms and biological pathways predicted by ClueGO. The list of conserved miRNAs predicted to target these genes was identified in TargetScanHuman^33^. To reconstruct the miRNA-mRNA regulatory network, the expression intensity of each miRNA and their respective targets was extracted from the normalized expression data of the Brazilian cohort of pterygium. Pairwise correlation was computed between miRNA and their targets. miRNAs that have a negative correlation (R equal or less than -0.5) with at least one target were selected for the network reconstruction. Network visualization was performed in Cytoscape^36^ software considering miRNA and mRNA as source and target nodes respectively.

## Results

### Collected data

Nine participants were included, being 6 males (aged 29–70 years old) and 3 females (aged 27–48 years old). The pterygia were classified as grade 1 in 1 case, grade 2 in 4 cases, grade 3 in 3 cases and grade 4 in 1 case. According to their biomicroscopic aspect, there were 2 atrophic and 7 active pterygia. Demographic information of the participants is presented in Table 1.

**Table 1:** Demographic information of the participants.

Participants	Age	Gender	Grade	Biomicroscopic aspect
1	45	Female	2	Active
2	53	Male	2	Atrophic
3	34	Male	1	Active
4	70	Male	3	Atrophic
5	27	Female	2	Active
6	48	Female	3	Active
7	35	Male	2	Active
8	51	Male	4	Active
9	29	Male	3	Active

#### Evaluation of genes that are divergently expressed between fleshy and atrophic pterygium

**Figure 1:**
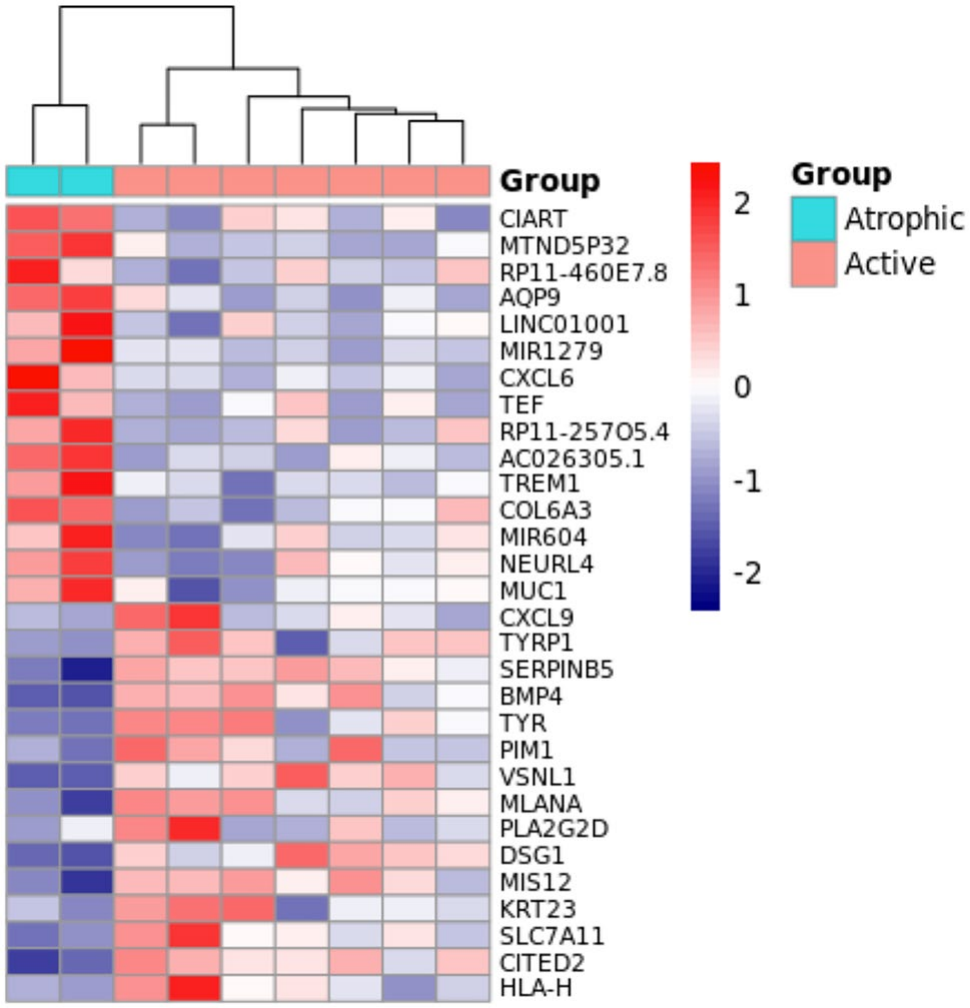
Heatmap plot of top 30 differentially expressed genes from the comparison of active pterygium with atrophic pterygium filtered by the fold change. The pattern of gene expression shows two different unsupervised clusters indicating a divergent expression pattern between these two clinical aspects. High expression is indicated in red and low expression is indicated in blue.

We evaluated the differential expression comparing active pterygium with atrophic pterygium using data generated from the Brazilian cohort. This analysis identified 219 up-regulated and 92 down-regulated genes. The pattern of gene expression generated with top 30 DE genes is presented as heatmap in Fig. 1. The top 10 up- and down-regulated genes and their main biological processes are presented in Table 2.

**Table 2:** List of top 10 up- and down-regulated genes in active *vs* atrophic pterygium and their associated biological processes.

DE	Fold change	*p* value	AUC	Main biological process
**Up-regulated**				
*CXCL9*	126.36	0.0003	0.86	Immune and inflammatory response
*TYRP1*	8.76	0.0065	0.86	Aldosterone synthesis and secretion
*SERPINB5*	7.29	0.0002	1.00	Angiogenesis
*BMP4*	7.04	0.0006	1.00	Degradation of the extracellular matrix
*TYR*	6.95	0.0006	1.00	(S)-reticuline biosynthesis and Tyrosine metabolism
*PIM1*	6.78	0.0075	0.93	Cell growth. differentiation and apoptosis
*VSNL1*	6.28	0.0016	1.00	Modulation of intracellular signaling pathways
*MLANA*	5.64	0.0007	1.00	NF-kappaB Signaling
*PLA2G2D*	5.44	0.0043	0.71	Targets extracellular lipids; anti-inflammatory and immunosuppressive functions
*DSG1*	5.35	0.0002	1.00	Keratinization
**Down-regulated**				
*CIART*	0.03	0.0032	1.00	Circadian rythm
*AQP9*	0.16	0.0001	1.00	Transporter activity and glycerol channel activity
*LINC01001*	0.17	0.0091	1.00	*
*mir1279*	0.18	0.0005	1.00	#
*CXCL6*	0.18	0.0027	1.00	Chemotactic and angiogenic properties
*TEF*	0.19	0.0083	1.00	Circadian rythm
*TREM1*	0.21	0.0024	1.00	Cell surface interactions at the vascular wall
*COL6A3*	0.22	0.0025	1.00	Integrin Pathway and Collagen chain trimerization
*mir604*	0.23	0.0009	1.00	#
*NEURL4*	0.23	0.0004	1.00	Ubiquitination and proteasome-dependent degradation

*Long non coding RNA; # miRNA.

#### Studies included in the meta-analysis

**Figure 2:**
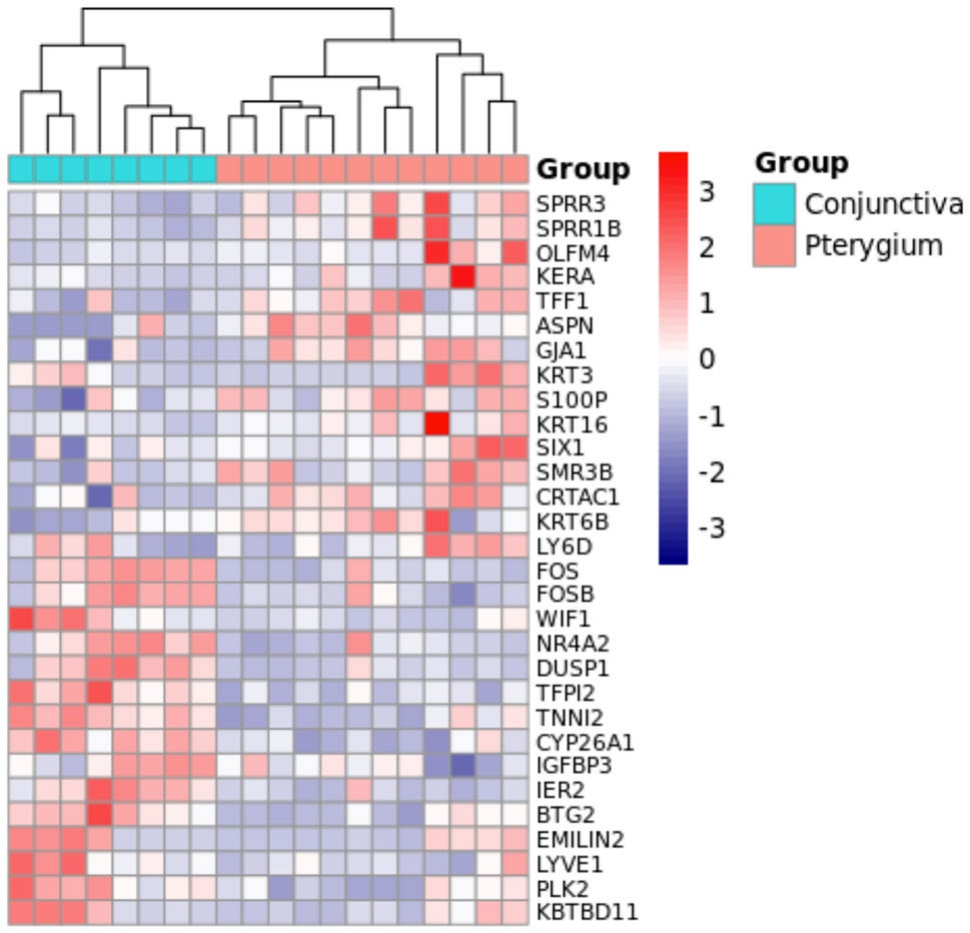
Gene expression pattern of conjunctiva and pterygium from the meta-analysis. The heatmap was constructed using the top 30 differentially expressed genes (15 up- and 15 down-regulated). The dendrogram indicates two clusters stratified using hierarchical clustering. Expression pattern was rescaled using a list of human housekeeping genes from HRT Atlas database^44^. High expression is indicated in red and low expression is indicated in blue.

Three studies (GSE2513, GSE51995 and GSE83627) fulfilled the inclusion and exclusion criteria described in methods section of which two datasets (GSE51995 and GSE83627) were identified as duplicated datasets by PCA analysis (Fig. 2; 8 samples in each dataset). An exploratory analysis also confirms the presence of 8 duplicates based on the comparison of the normalized expression of that datasets. Therefore, GSE83627 was removed from the meta-analysis. The studies of the final analysis include data from 8 conjunctiva samples and 12 pterygium samples.

#### Differential expression of pterygium

The meta-analysis identified 154 up-regulated and 58 down-regulated genes (Table 3). The top 10 up- and down-regulated genes are presented in Table 3. A heat map visualization of the expression pattern of the top 30 DE genes identified from the meta-analysis showed a stratification of conjunctiva and pterygium samples in two different clusters (Fig. 2).

**Table 3:** Top differentially expressed genes identified in the meta-analysis.

Genes	Fold-change in individual studies (log2 FC)		Meta-analysis results		
	GSE2513	GSE51995	Ave log2(FC)	FC	FDR
**Up-regulated genes**					
SPRR3	2.16	3.78	2.97	7.85	< 0.0001
SPRR1B	1.91	3.23	2.57	5.94	< 0.0001
OLFM4	0.23	4.61	2.42	5.35	< 0.0001
KERA	0.53	4.02	2.28	4.85	< 0.0001
TFF1	2.49	1.66	2.07	4.21	< 0.0001
ASPN	1.39	2.37	1.88	3.67	< 0.0001
GJA1	1.04	2.58	1.81	3.51	< 0.0001
KRT3	0.09	3.51	1.80	3.49	< 0.0001
S100P	1.15	2.44	1.79	3.47	< 0.0001
KRT16	0.74	2.76	1.75	3.36	< 0.0001
**Down-regulated genes**					
FOS	− 3.84	− 3.21	− 3.53	0.09	< 0.0001
FOSB	− 2.39	− 2.73	− 2.56	0.17	< 0.0001
WIF1	− 0.49	− 4.06	− 2.28	0.21	< 0.0001
NR4A2	− 2.21	− 1.68	− 1.94	0.26	< 0.0001
DUSP1	− 1.82	− 1.85	− 1.84	0.28	< 0.0001
TFPI2	− 0.84	− 2.47	− 1.65	0.32	< 0.0001
TNNI2	− 1.46	− 1.74	− 1.60	0.33	< 0.0001
CYP26A1	− 1.21	− 1.58	− 1.39	0.38	< 0.0001
IGFBP3	− 1.21	− 1.43	− 1.32	0.40	< 0.0001
IER2	− 1.12	− 1.47	− 1.29	0.41	< 0.0001
BTG2	− 1.17	− 1.38	− 1.28	0.41	< 0.0001

Genes were ranked according to the fold change. FC: Fold-change; Ave log2(FC): Base 2 logarithmic scale of average FC; FDR: False Discovery Rate. Ave log2(FC) is expressed as arithmetric mean log2(FC) across studies.

#### Pterygium up-regulated gene signature highlights a prominent role of extracellular matrix degradation and remodeling

**Figure 3:**
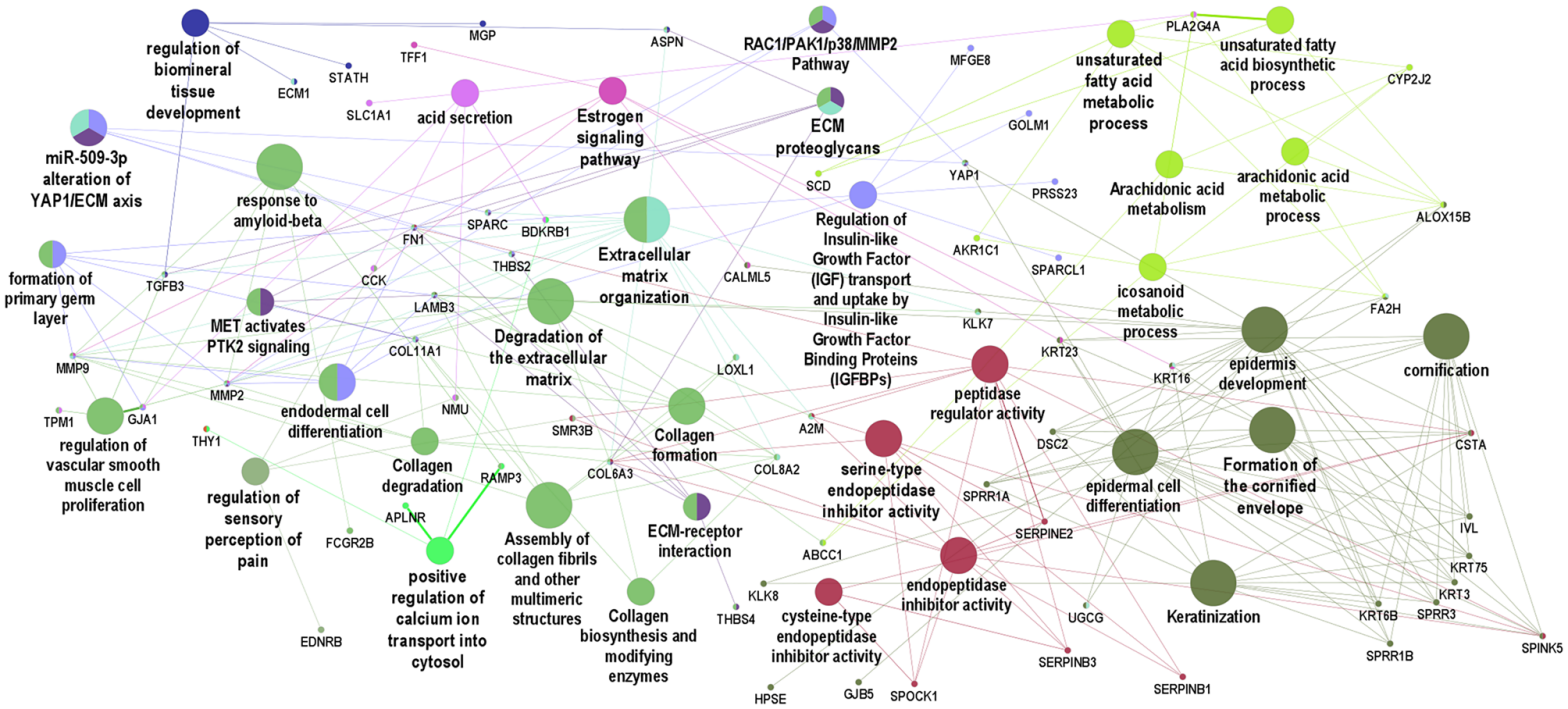
Functional analysis of up and down regulated genes in pterygium. The main pathways that are altered in patients with pterygium are indicated by colored nodes. GSA terms are interconnected with their shared genes. Close related terms are indicated by more than one color. GSA: Gene Set Analysis.

To gain further insights into the biological mechanisms associated with the expression pattern observed in the meta-analysis, a gene set analysis was performed with ClueGO using the full list of the up-regulated genes. This tool clusters biological pathways and gene ontology terms that participate in the same biological function, thereby showing the top significant non-redundant biological pathways and gene ontology terms (Fig. 3). Pathways associated with the remodeling of extracellular matrix were overrepresented in this gene set analysis. The following groups were also represented in the network: serine-type endopeptidase activity, formation of cornified envelope, estrogen signaling pathway, unsaturated fatty acid metabolic process, regulation of sensory perception of pain, positive regulation of calcium ion transport into cytosol and regulation of biomineral tissue development. ClueGO also enables the visualization of gene interactions between different gene ontology and biological pathways. Based on network connectivity, *MMP2, FN1, COL11A1, LAMB3, THBS2* and *YAP1* were predicted as hub genes and might play prominent role in the organization of this network. Interestingly, these genes show a tight relationship between extracellular matrix remodeling terms and other pathways and ontology terms (Fig. 3).

#### Prediction of the interaction networks of the DE genes and miRNAs in pterygium

**Figure 4:**
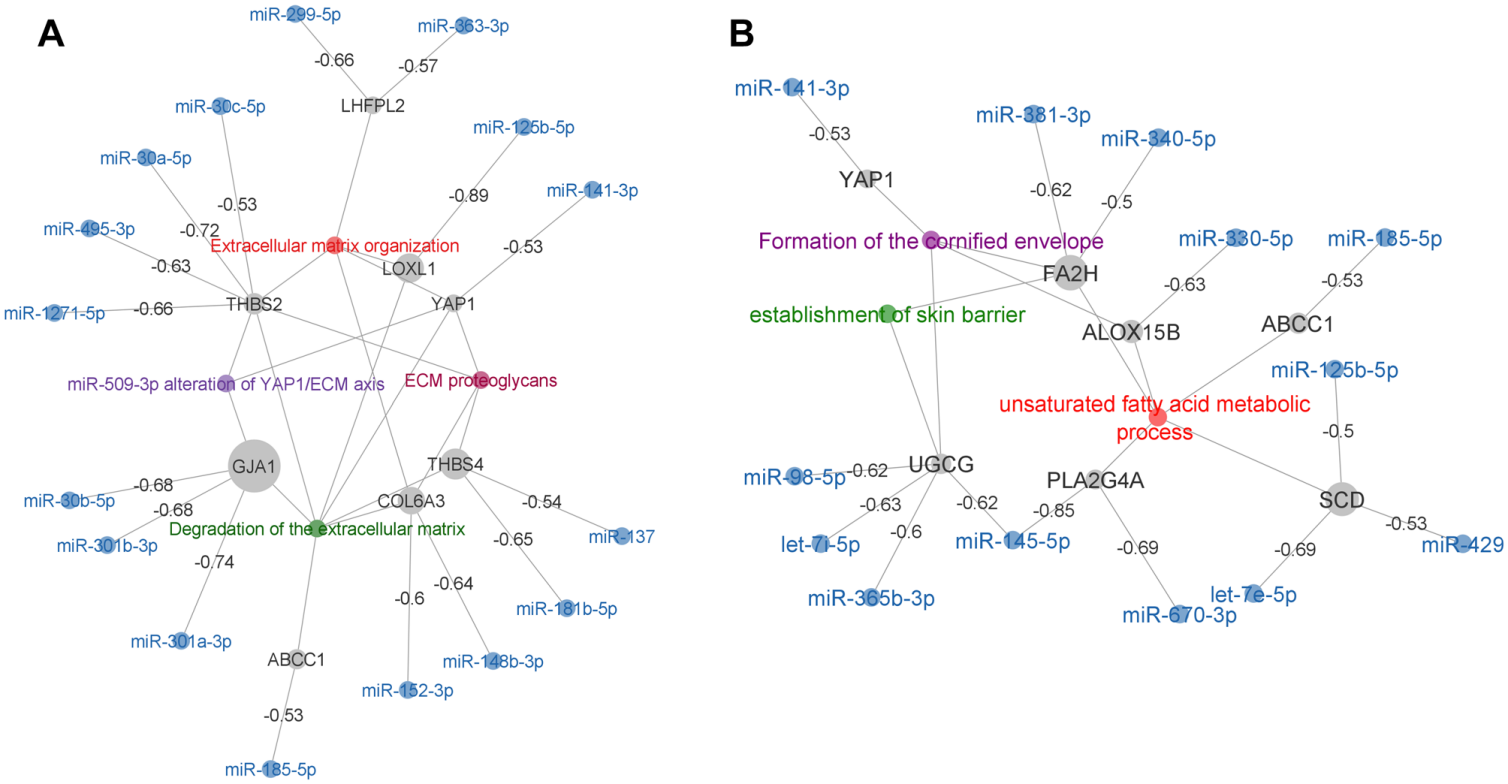
miRNA-mRNA targeting networks generated from the Brazilian cohort. miRNA-mRNA interaction was predicted using TargetScanHuman database. Pairwise correlation was computed between miRNA expression and the expression of the set of up-regulated genes presented in the gene ontology network generated in Fig. 2A. Only miRNA and genes which have negative correlation (less than − 0.50) were included. Regulatory networks associated with extracellular matrix remodeling are clustered (**A**). The second cluster (**B**) involves 3 different terms including the formation of the cornified envelope, the establishment of skin barrier and unsaturated fatty acid metabolic process. Genes are represented in grey dots and dot size is proportional to the level of expression; miRNA are represented in blue and edges are labeled with the correlation level between miRNA and genes.

To further explore the mechanism of regulation underlying the biological processes identified in the meta-analysis, we reconstructed the miRNA-mRNA target prediction network based on the set of up-regulated genes presented in the gene ontology and biological pathways network (Fig. 3). This strategy allows the identification of 17 target genes negatively correlated with their interacting miRNAs from the Brazilian cohort expression data (Fig. 4). Based on network connectivity analysis we identified two main clusters (Fig. 4A,B). Once again, the first cluster is associated with 4 enriched GSA terms involving extracellular matrix remodeling mechanisms (Fig. 4A). The second cluster involves three biological processes: (1) formation of the cornified envelope, (2) establishment of skin barrier and (3) unsaturated fatty acid metabolic process (Fig. 4B).

#### Down-regulated genes in the pterygium meta-analysis are associated with metabolic and regulation pathways

**Figure 5:**
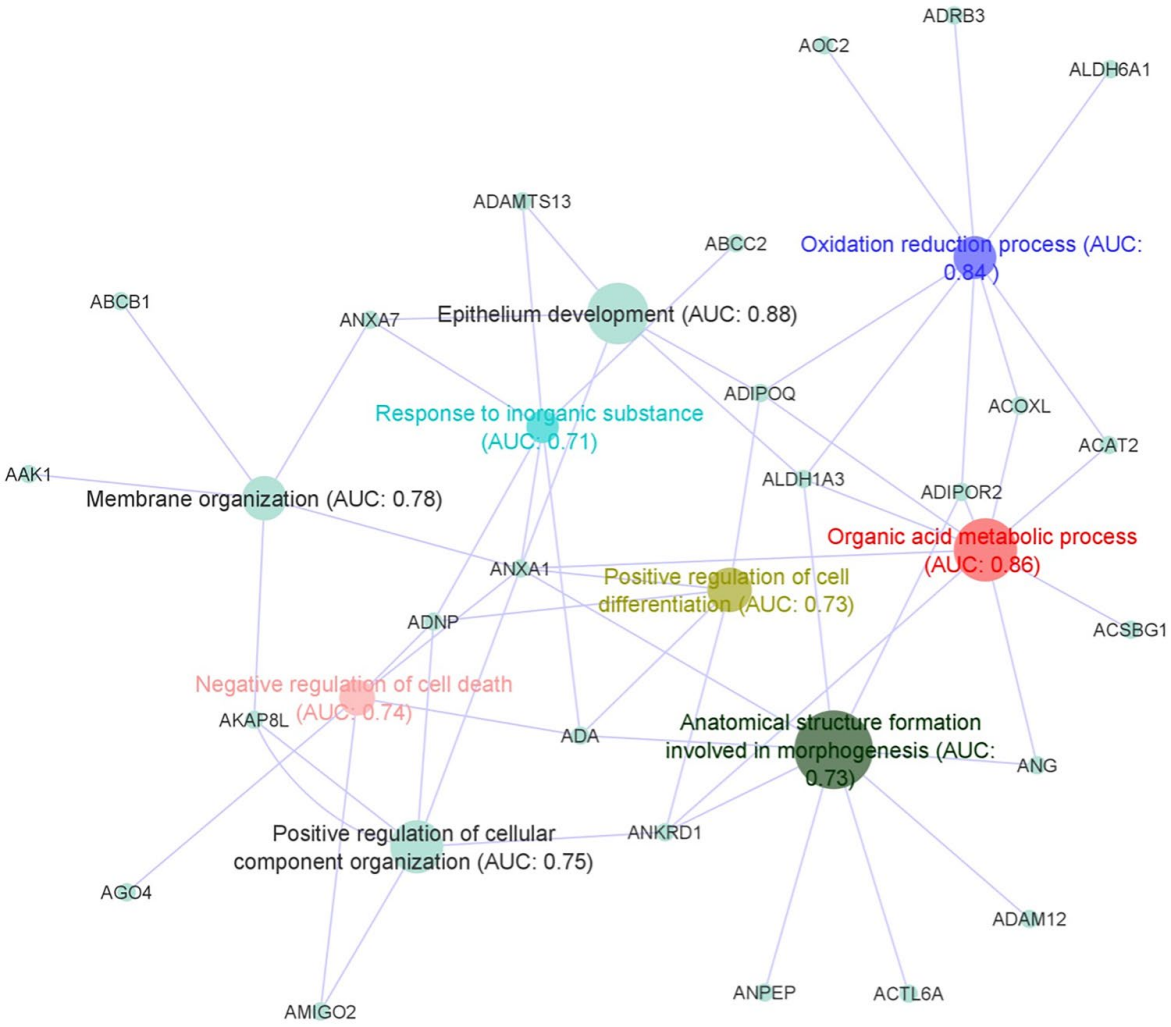
Functional analysis of the down-regulated genes identified in the meta-analysis based on FAIME algorithm. Main down-regulated pathways are indicated by colored nodes. Pathways were selected based on the FAIME scores computed using the expression of down-regulated genes (grey dots). AUC was used to evaluate the discriminatory capacity of each enriched term for a binary classification (pterygium vs. control). FAIME: Functional Analysis of Individual Microarray/RNAseq Expression; AUC: Area Under the curve.

In order to study the mechanisms underlying the down-regulated pattern observed in the meta-analysis, we next performed a functional analysis based on FAIME algorithm that attributes at a single sample level a score to each predicted term or biological process. This analysis shows mainly GSA terms associated with metabolic and regulation pathways (Fig. 5) ranked by their discriminatory capacity to classify pterygium and control in different clusters (AUC equal or more than 0.7). Gene interactions between different gene ontology and biological pathways are shown in Fig. 5.

## Discussion

Despite being a common disease of the ocular surface, the pterygium exact etiopathogenesis is not yet fully understood. For this reason, a meta-analysis of transcriptomic datasets deposited in public repositories was performed in order to generate new biological insights about its pathogenic mechanisms. Furthermore, gene expression data was obtained from 9 Brazilian patients and later correlated with data from the meta-analysis.

In the Brazilian cohort, 66.7% of the patients were males, 44.4% had pterygium grade 2 and 77.8% had active pterygium. Demographic and epidemiological characteristics of this cohort are in accordance with previously described features of patients with pterygium in our region, as described by Artioli-Schelini et al., that found that most of the patients had active and grade 2 pterygia^3^.

In our meta-analysis, we found 212 DEGs, being 154 up-regulated and 58 down-regulated genes. Amongst the top up-regulated genes, *SPRR3, SPRR1B, OLFM4, KERA, TFF1, ASPN, GJA1, KRT3, S100P* and *KRT16* were the most significantly differentially expressed genes. On the other side, amongst the top down-regulated genes, *FOS, FOSB, WIF1, NR4A2, DUSP1, TFPI2, TNNI2, CYP26A1, IGFBP3, IER2* and *BTG2* were the most significantly differentially expressed ones. As in previous studies, we found that genes associated with wound healing response and extracellular matrix were the most predominantly overexpressed^46,47^. *SPRR3* and *SPRR1B* encode envelope proteins of keratinocytes, being part of the cornification process that culminates with the formation of a keratinized cell layer^46,47^. In the conjunctiva, this occurs in response to desiccating stress, which is in accordance with known risk factors of pterygium, such as exposure to wind and high temperatures. *KERA* encodes a keratan sulfate proteoglycan that is involved in corneal transparency and its up-regulation probably leads to extracellular matrix changes. *TFF1* is a gene that encodes small protease-resistant peptides, which are secreted by goblet cells in the conjunctiva and are involved in triggering wound-healing responses^48^. *ASPN* encodes a protein that is member of the small leucine-rich proteoglycan family, also being part of extracellular matrix. *KRT3* and *KRT16* encode keratin 3 and 16, respectively, which are protein members of the keratin family, associated with epithelial cell differentiation and cornification. S100 calcium binding protein C is involved in regulation of cell cycle progression, differentiation and also wound healing mechanisms, possibly having a role in the pathogenesis of pterygium through epithelial wound repair and response to stress^49^. *TFPI2* encodes an inhibitory protein that inhibits plasmin, trypsin and other serine proteases, possibly playing a role in pterygium pathogenesis once it is involved in extracellular matrix modulation.

A meta-analysis performed by Xu and colleagues, based on three datasets (GSE2513, GSE51995 and GSE83627) available at the Gene Expression Omnibus public repository, disclosed upregulation of other genes in addition to the ones found in our meta-analysis, including FN1, a key molecule in the extracellular matrix, and PI3, involved in apoptosis, focal adhesion and extracellular matrix-receptor interaction^47^. In a study using gene microarray performed by Tong and colleagues, several pathways were also significantly affected in pterygium in comparison with uninvolved conjunctiva. Gene expression was predominantly of wound healing pattern, with upregulated genes encoding for extracellular matrix (ECM), structural and adhesion molecules, including fibronectin (FN1), CEACAM5 (CEA), CD24, SPARC, MSMB and TFF1^46^. In addition, Chen and colleagues performed RNA sequencing experiments on clinical pterygium tissues and normal conjunctival tissues and identified 200 upregulated DGEs and 139 downregulated DGEs. The upregulated genes were mainly associated with ECM and with cell adhesion and migration and included: FN1; ECM1, which encodes extracellular matrix protein 1; IQGAP2, associated with cell adhesion and cytoskeletal organization; GADD34, a growth cycle protein that is induced by cell stress; and CXCL12, an angiogenic chemokine^50^. Biological pathways overrepresented in the gene set analysis were highly related to remodeling of extracellular matrix, including its organization, degradation, associated proteoglicans and regulation of biomineral tissue development. It was evidenced not only in the network of up-regulated genes, but also in the correlation between this gene set with the miRNA expression obtained from the Brazilian cohort. Other pathways also represented in the network and in the correlation with miRNA expression included formation of cornified envelope, establishment of skin barrier and unsaturated fatty acid metabolic process, which are associated with wound healing response.

Amongst the genes correlated with miRNA expression from the Brazilian cohort, GJA1 encodes a protein called connexin 43, a component of gap junctions, COL6A3 encodes one of the chains of type VI collagen, a component of connective tissues, and THBS4 and THBS2 encode for members of the thrombospondin family, which are adhesive glycoproteins that mediate cell-to-cell and cell-to-matrix interactions. YAP1 is a gene known to play a role in the development and progression of multiple cancers as a transcriptional regulator of a signaling pathway. LOXL1 encodes a protein essential to the biogenesis of connective tissue, while LHFPL2 is a member of the superfamily of tetraspan transmembrane protein encoding genes.

Also, regarding the genes correlated with miRNA expression obtained from the Brazilian cohort, pathways involved with unsaturated fatty acid metabolic processes were found to be differentially expressed in pterygium. This supports other studies findings in which pterygium fibroblasts were found to have an increased activity of intracellular cholesterol metabolism and high expression of LDL receptors^51–53^. ALOX15B encodes a protein involved in the production of fatty acid hydroperoxides and FA2H, a protein that catalyzes the synthesis of a subset of sphingolipids. ABCC1 is involved in the transport across extra and intra-cellular membranes and UGCG, in the biosynthesis of glycosphingolipids, which are membrane components. PLA2G4A encodes a cytosolic phospholipase A2, an enzyme that catalyzes the hydrolysis of membrane phospholipids to release arachidonic acid. SCD encodes an enzyme involved in fatty acid biosynthesis, primarily the synthesis of oleic acid.

Also reinforcing previous studies findings, increased cellular proliferation and reduced apoptosis appear to be involved in pterygium development^47^, highlighting similarities between pterygium and neoplasia. Clinical evidence of tumoral behavior include fibrovascular proliferation, corneal invasion, and high recurrence rate after surgical excision. The reduced recurrence rate of pterygia with the application of mitomycin-C, 5-fluorouracil or cyclosporine during or after surgery supports this finding, as does the fact that the main risk factor for pterygium formation is UV light exposure, equivalently to the most common skin tumors^54^.

OLFM4 consists of an antiapoptotic factor that promotes tumor growth and also an extracellular matrix glycoprotein that facilitates cell adhesion. In pterygium’s pathogenesis, it could be associated an uncontrolled tissue growth. GJA1, a member of the connexin gene family, encodes the major protein of gap junctions and is related to cell signaling, apoptotic processes and chronic inflammatory processes. The *FOS* gene family consists of 4 members, which are *FOS, FOSB, FOSL1* and *FOSL2*. The proteins encoded by these genes are leucine zipper proteins involved in regulation of cell proliferation, differentiation and transformation. In addition, these proteins play a role in cellular response to reactive oxygen species and in inflammatory response^46^. *WIF1* consists of a tumor suppressor gene that is epigenetically silenced in various cancers. *NR4A2* encodes a nuclear receptor of the steroid-thyroid hormone-retinoid receptor family and may be involved in pterygium once it is associated with cellular response to oxidative stress and regulation of apoptotic signaling pathway. *DUSP1* encodes a protein that plays an important role in the cellular response to environmental stress and in the negative regulation of cellular proliferation. *TNNI2* encodes a component of the troponin complex, present in corneal epithelium, acting as an inhibitor of angiogenesis and tumor suppressor. *CYP26A1* encodes a member of the cytochrome P450 superfamily of enzymes, involved with regulation of retinoic acid receptor pathway and response to vitamin A. IGFBP3, one of the insulin-like growth factor binding proteins, is involved in apoptotic processes and regulation of cell population proliferation. *IER2* is an immediate early gene involved in cell motility and response to fibroblast growth factor. Lastly, the protein encoded by *BTG2* appears to have antiproliferative properties, being associated with DNA damage response and repair and having a function of negatively regulating the mitotic cell cycle.

In Tong and colleagues’ study, genes encoding for apoptosis (*TGM2, IGFBP3* and *DUSP1*) and stress-inducible transcription regulator genes (*ATF3, BTG2, EGR1, ERG2, FOS, JUN, NR4A1* and *NR4A2*) were down-regulated in pterygium, reinforcing the over-proliferative tendency in pterygium^46^. Downregulated genes in Chen and colleagues’ study, in its turn, included *LCN1*, a member of the lipocalin family, *LTF*, a component of the innate immune system, and *SCGB2A1*^50^. Biological functions found to be down-regulated in pterygium in comparison with normal conjunctiva included regulation of cell differentiation and cellular component organization, oxidation–reduction processes and negative regulation of cell death. This reinforces the similarities between pterygium and tumor growth^9^, although differences still exist. Amongst the differences between the two conditions, the fact that extracellular matrix remodeling is the main mechanism found in pterygium’s pathogenesis, and not cellular proliferation, is one of them. The main histologic findings in pterygium’s specimens include squamous metaplasia, hyperplasia of goblet cells, underlying disrupted Bowman’s layer, stromal fibroblasts and vessels, altered extracellular matrix with accumulation of collagen and elastin fibers, and inflammatory infiltration^55^. As shown in previous studies, histopathology only rarely discloses neoplasia or moderate/severe atypia in excised pterygia^55–57^.

Finally, we could determine differences in gene expression between atrophic and active pterygium, leading to the conclusion that distinct biological pathways may be differently combined in terms of up and down-regulation to result in certain phenotypic characteristics of pterygium. In active pterygium, genes involved with immune and inflammatory response (*CXCL9* and *PLA2G2D*), angiogenesis (*SERPINB5* and *BM4*), keratinization (*DSG1*), response to UV light (*TYR*) and negative regulation of apoptotic process (*PIM1*) are up regulated in relation to atrophic pterygium. On the other side, genes involved with negative regulation of transcription (*CIART*), response to osmotic stress (*AQP9*), chemotaxis (*CXCL6*), intracellular signal transduction (*TREM1*), extracellular matrix organization (*COL6A3*) and protein ubiquitination (*NEURL4*) are down regulated in relation to atrophic pterygium.

Limitations of the study included the Brazilian sample size, which was not large, in addition to an absence of healthy conjunctival samples to compare with pterygium samples. Consequently, these samples were not included in the analysis of up and down regulated genes of the main meta-analysis, which was based on already available datasets. Brazilian data was used for the reconstruction of the miRNA-mRNA regulatory network and for a transcriptomic analysis in which active and atrophic pterygia were compared. Even though the sample size of this analysis was limited, the top ranked genes made it possible to stratify active and atrophic pterygium in different clusters using unsupervised clustering method as shown in Fig. 1.

In conclusion, reinforcing previous studies, we identified crucial pathways in the pathophysiology of pterygium, with the main ones being related to extracellular matrix remodeling and dysregulated wound healing response. Furthermore, different pathways involved in the pathogenesis of pterygium were evidenced to be related to different phenotypic characteristics of this condition, which may also provide some clarity in our understanding of this condition’s pathophysiology. Thus, we believe this study enriches our understanding of molecular mechanisms involved with pterygium, providing insights that may contribute for the future development of new therapeutic targets for its management.
